# Intermittent fasting induces chronic changes in the hepatic gene expression of Red Jungle Fowl (Gallus gallus)

**DOI:** 10.1186/s12864-022-08533-5

**Published:** 2022-04-14

**Authors:** Caroline Lindholm, Petros Batakis, Jordi Altimiras, John Lees

**Affiliations:** 1 AVIAN Behavioural Genomics and Physiology Group, IFM, Linköping University, 58183 Linköping, Sweden; 2 Present Address: Laboratory of Organic Electronics, ITN, Linköping University, 60174 Norrköping, Sweden; 3 Present Address: Benaki Phytopathological Institute, Athens, Greece; 4 Present Address: Department of Organismal Biology, Uppsala University, 75236 Uppsala, Sweden

**Keywords:** Gallus gallus, Intermittent fasting, Liver transcriptomics, Microarray, Red junglefowl, Skip-a-day

## Abstract

**Background:**

Intermittent fasting (IF), the implementation of fasting periods of at least 12 consecutive hours on a daily to weekly basis, has received a lot of attention in recent years for imparting the life-prolonging and health-promoting effects of caloric restriction with no or only moderate actual restriction of caloric intake. IF is also widely practiced in the rearing of broiler breeders, the parent stock of meat-type chickens, who require strict feed restriction regimens to prevent the serious health problems associated with their intense appetites. Although intermittent fasting has been extensively used in this context to reduce feed competition and its resulting stress, the potential of IF in chickens as an alternative and complementary model to rodents has received less investigation. In both mammals and birds, the liver is a key component of the metabolic response to IF, responding to variations in energy balance. Here we use a microarray analysis to examine the liver transcriptomics of wild-type Red Jungle Fowl chickens fed either ad libitum, chronically restricted to around 70% of ad libitum daily or intermittently fasted (IF) on a 2:1 (2 days fed, 1 day fasted) schedule without actual caloric restriction. As red junglefowl are ancestral to domestic chicken breeds, these data serve as a baseline to which existing and future transcriptomic results from farmed birds such as broiler breeders can be compared.

**Results:**

We find large effects of feeding regimen on liver transcriptomics, with most of the affected genes relating to energy metabolism. A cluster analysis shows that IF is associated with large and reciprocal changes in genes related to lipid and carbohydrate metabolism, but also chronic changes in genes related to amino acid metabolism (generally down-regulated) and cell cycle progression (generally up-regulated). The overall transcription pattern appears to be one of promoting high proliferative plasticity in response to fluctuations in available energy substrates. A small number of inflammation-related genes also show chronically changed expression profiles, as does one circadian rhythm gene.

**Conclusions:**

The increase in proliferative potential suggested by the gene expression changes reported here indicates that birds and mammals respond similarly to intermittent fasting practices. Our findings therefore suggest that the health benefits of periodic caloric restriction are ubiquitous and not restricted to mammals alone. Whether a common fundamental mechanism, for example involving leptin, underpins these benefits remains to be elucidated.

**Supplementary Information:**

The online version contains supplementary material available at 10.1186/s12864-022-08533-5.

## Background

Intermittent fasting (IF) has gained a lot of attention in recent years for imparting many of the health benefits associated with caloric restriction, without actually restricting total caloric intake [1]. IF is typically defined as eating patterns involving at least 12 h of continuous fasting on a daily to weekly basis [2]. Reported health benefits of IF include weight loss with maintained lean mass [3], improved insulin sensitivity [3] and protection from age-related [4] as well as pathology-induced cognitive decline [5]. Many of the health benefits associated with IF have been attributed to the metabolic switching between a glucose-based and a ketone-based energy metabolism that comes about as a result of hepatic glycogen stores being depleted within 12–36 h after the last consumed meal [2]. This switch is driven by metabolic processes in the liver, where energy originally supplied through glycogenolysis is replaced by that from ketogenesis and gluconeogenesis, and will eventually induce changes in energy substrate preference in peripheral tissues [2, 6].

As well as numerous experimental models of IF in mammals, IF is also utilized in industry in the rearing of “broiler breeder” chickens, often under the name “skip-a-day” feeding [7]. Feed restriction is routinely used in the rearing and maintenance of this meat-type parent stock to combat long-term health problems associated with the extreme appetites that these animals are bred for [7, 8]. Intermittent fasting regimens are commonly used in this setting to increase portion sizes and to reduce food competition and the resulting meal-time stress and aggression [7, 8]. Despite this, IF in farmed chickens is widely considered detrimental to animal health and welfare [7] and has even been linked to increased adiposity in some studies [9, 10].

One reason for the seemingly opposing views on IF in the mammalian versus poultry literature may be that meat-type chickens cannot be fed ad libitum (AL) over longer time periods without developing severe health problems, fertility problems, and excessive mortality [11, 12]. Even in “skip-a-day” feeding regimens, IF must be combined with overall caloric restriction to around 30% of AL intake. In this paper, we aim to better understand the response to IF in poultry by instead using the Red Jungle Fowl (RJF), a wild-type chicken which is ancestral to domestic chickens, is not prone to diet-induced obesity and remains healthy on an AL diet. We raised a cohort of young RJF on either AL feeding, chronic daily restriction (CR) to around 70% of AL intake or an IF diet (two feeding days at 150% of daily AL intake offered followed by a fasting day) from 2–5 weeks of age. Birds fed according to the CR and IF feeding regimens showed similar growth rates, although IF-fed birds had a consistently higher feed intake as previously reported [13]. Reduced body weights with maintained feed intake have also been reported in mice [14], but are not always seen [15]. It is well known that the liver is central to the primary metabolic response to dietary changes in chickens [16], and shows large fluctuations in mass, glycogen content and lipid content under IF conditions in both RJF [13] and broiler breeders [10, 17] just as in mice [18]. Concomitant with these overt physiological effects are large effects on hepatic transcription. Although the transcriptomic response to acute fasting in chicken liver has been studied [19], we are not aware of any study that has examined this transcriptomic response to IF in chickens, except for a study on a subset of hepatic genes [17].

In this study, intermittently fasted chickens were sampled at two different time points: the second consecutive day of feeding (F2) and the fasting day (SK), which were expected to represent the two extremes of the metabolic switch. We subsequently perform cluster and pathway analyses on the liver transcriptome at these timepoints and compare them to both liver physiology traits (liver mass, lipid content, glycogen content) and appetite-associated gene expression in the arcuate nucleus (ArcN). We also take a special interest in two types of expression patterns in IF chickens: 1) cyclically switching expression patterns where F2 and SK expression levels are significantly different as these may be associated with metabolic switching and its health benefits or drawbacks, and 2) chronic changes in gene expression where F2 and SK expression are significantly different from the AL-fed controls in a consistent direction which are potentially related to long-term health effects of IF.

## Results

**Fig. 1:**
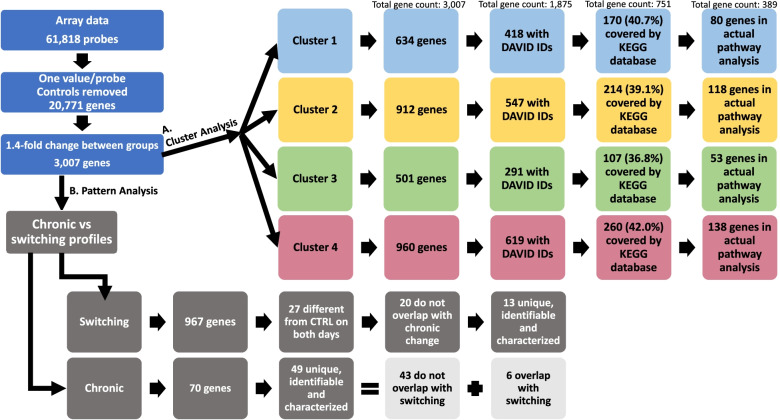
Flow of data in the analysis. The microarray contained 61,818 probes corresponding to 20,771 genes. A total of 3,007 of these met the criteria of at least 1.4-fold change between groups and were subjected to (**A**) cluster and KEGG pathway analyses, as well as (**B**) a pattern analysis looking for genes showing switching and chronically changed (compared to AL) expression patterns

The flow of microarray data analysis is presented in Fig. 1. In brief, data from 61,818 microarray probes corresponded to 20,771 probe sets. Of these, 3,007 met the criteria of *P* ≤ 0.01 and a fold change ≥ 1.4, the latter chosen for comparability with previously published chicken liver transcriptomics [19]. The number of up- and down-regulated genes were roughly equal in all comparisons, ranging from 124 up- and 157 down-regulated genes (comparing AL and F2), to 798 up- and 786 down-regulated (comparing F2 and SK) (Additional Table 1). In total, 1,675 genes showed upregulation and 1,630 showed downregulation in at least one treatment comparison with some genes showing both patterns depending on which groups were being compared (Additional figure 1).

### Cluster and pathway analyses

**Fig. 2:**
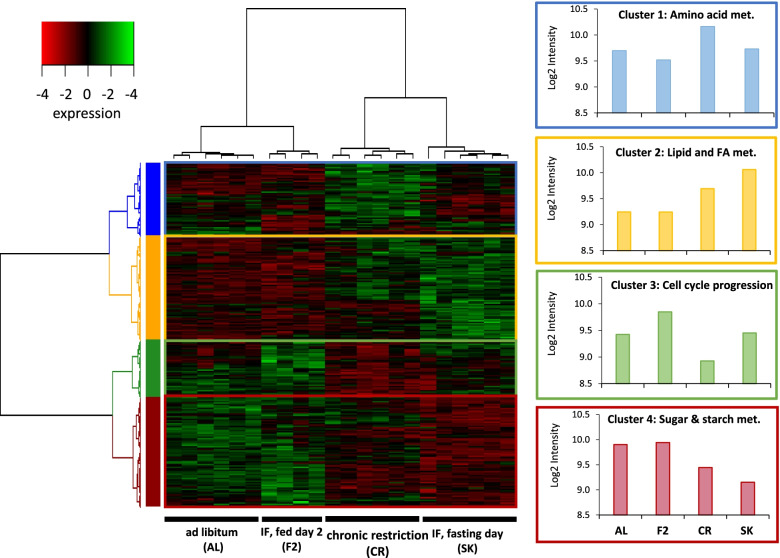
Heatmap of microarray expression data with clusters. The data was clustered into four groups, successfully identifying the four feeding conditions (columns). Overall expression of the four gene clusters (rows) are shown in color-coded panels on the right. Headings for the four clusters have been chosen to represent most of the pathway terms as presented in detail in Fig. 4. The heatmap was generated using the Bioconductor package in R

**Fig. 3:**
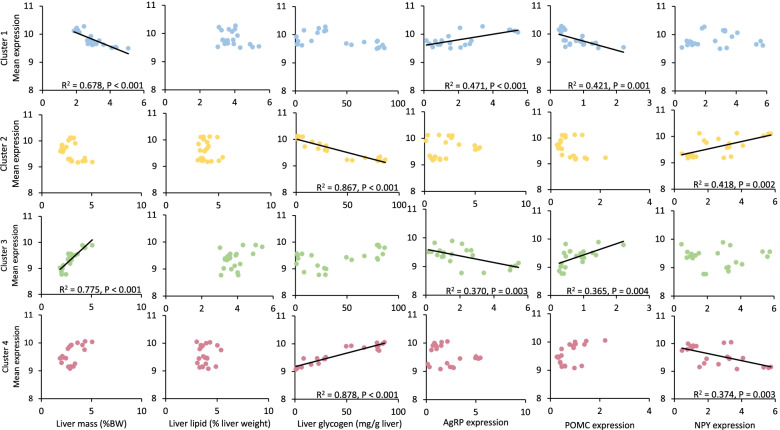
Correlation of mean cluster expression with other measured traits. The mean expression of each clustered underwent correlation analysis with each of seven physiological traits (body mass, absolute liver mass, relative liver mass, total liver lipids, liver lipid concentration, total liver glycogen, liver glycogen concentration) and the expression of three appetite-regulated genes in the arcuate nucleus. Correlations were considered significant at *P* < 0.005 (Bonferroni-corrected from *P* < 0.05)

The gene set underwent a cluster analysis, sorting genes into four clusters (Fig. 2, heatmap rows). The clustering analysis successfully differentiated between the four feeding treatments, grouping the two fully fed states (AL and F2) together and the underfed states of chronic restriction and fasting (CR and SK) together. The gene clusters were also split into two cluster pairs, with clusters 1 and 2 (634 and 912 genes respectively) showing general down-regulation in the fully fed states. Cluster 1 was upregulated in CR but not in fasting, with cluster 2 showing a near-opposite pattern. Clusters 3 and 4 (501 and 960 genes respectively) instead showed general up-regulation in the fully fed states and were mostly down-regulated in CR. Cluster 4 was also down-regulated in SK, but cluster 3 was not. (Fig. 2). Correlational analyses were performed to compare the mean expression of each cluster to known parameters of liver physiology and ArcN expression of appetite-related genes (Fig. 3, Additional figure 2). Clusters 1 and 3 were both significantly correlated with relative liver mass (expressed as % body weight), and the ArcN expression of the appetitive agouti-related protein (AgRP) and the anorexic pro-opiomelanocortin (POMC) (Fig. 3). The correlational patterns were opposite with higher Cluster 1 expression in individuals with low relative liver mass, low POMC expression and high AgRP expression and the reversed situation for Cluster 3. Clusters 2 and 4 were instead correlated with hepatic glycogen reserves and ArcN expression of the consummatory orexigenic signal neuropeptide Y (NPY). Again, these two clusters showed opposite patterns with Cluster 2 expression showing negative correlation with glycogen concentration and positive correlation with NPY expression, and Cluster 4 the reverse (Fig. 3). No cluster was significantly associated with body mass after correction for multiple testing.

**Fig. 4:**
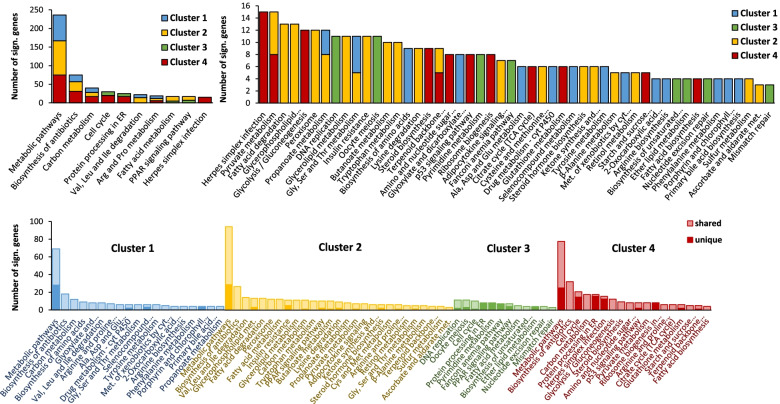
Results from the KEGG pathway analysis. Top row shows all the pathway terms in order of number of genes (the term “Herpes simplex infection” occurs in both graphs for scale) color-coded for the clusters which they appeared in. Bottom row shows each cluster separately divided into shared (ie. occurring in several of the pathways within the cluster) and unique (ie. only occurring in that one pathway) genes

Each cluster was also subjected to a KEGG analysis. Approximately 40% of the cluster genes were covered by the KEGG database and about half of those were included in the pathway analysis (Fig. 1). Clusters 1, 2 and 4 were heavily loaded with metabolic pathways, with the general “Metabolic pathways” category making up 36.1%, 27.0% and 27.0% of the transcripts respectively (Fig. 4). Of these, 40.6%, 28.3% and 30.7% respectively were exclusive to that category. Cluster 1 mainly consists of genes related to amino acid metabolism, cluster 2 is heavily loaded with lipid and fatty acid metabolism genes and cluster 4 is home to most transcripts related to the metabolism of sugars and starch. Cluster 3, which was by far the smallest cluster in the pathway analysis, was instead made up of genes related to DNA replication and cell cycle progression (Fig. 4). While many of the main terms highlighted in the pathway analysis are ambiguous and largely overlapping, some particularly interesting pathway terms worth noting include cell cycle (30 genes), PPAR signaling (17 genes) and insulin resistance (11 genes). Other pathway terms include “biosynthesis of antibiotics”, a set of 75 genes that entirely overlap with other pathways and are largely made up of genes involved in basic metabolic processes and “Herpes simplex infection” which contains a mostly unique collection of 15 genes involved in apoptosis (FADD, CASP8), inflammatory JAK/STAT signaling (SOCS3, JAK1, IFNGR2), transcriptional activation (TBPL1, ALYREF), splicing (SRSF2, SRSF3, LOC100859609) and protein synthesis (EIF2S1) as well as ERK signaling (FOS, JUN) an S-phase protein (SKP2) and a circadian regulator (CLOCK).

### Expression pattern profiling

In addition to the cluster-based analysis we performed a pattern analysis looking for genes with expression profiles of particular interest. The same 3,007 probe sets that were subjected to the cluster and KEGG pathway analyses were also examined for expression patterns that could be of certain interest in the context of metabolic switching. For this, we looked for two different expression patterns: either a pattern directly resembling the metabolic switch with F2 and SK being significantly different from each other, or a chronic change in expression (F2 and SK both significantly different from AL and changed in the same direction) which would be expected for mechanisms involved in long-term health effects of metabolic switching.

**Fig. 5:**
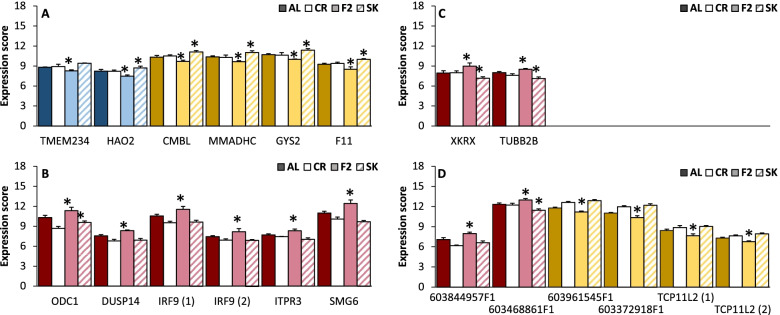
The expression of 18 genes showing a switching pattern under IF. The gene set is made up of (**A**) six characterized genes that are up-regulated in fasting (SK), (**B**-**C**) seven characterized genes that are down-regulated in fasting and (**D**) five uncharacterized genes. The expression of these genes is significantly different from AL on both F2 and SK days but in different directions, * indicates significant difference from CR. Colors indicate which cluster each gene appeared in in the cluster analysis

A total of 967 genes met the switching criteria with the expression of at least one of the IF days being significantly different from AL, although this includes some duplicates due to retired annotations (Fig. 1). For further analysis, we chose to restrict ourselves to the 27 genes that were significantly different from AL under both F2 and SK conditions (but in different directions). Of these, seven entries also met the criteria for chronic change and will be considered as part of that dataset. A total of 13 genes were found to be unique, identifiable and have mammalian orthologs that have been characterized; one more was identifiable but uncharacterized (TCP11L2) and four were unidentifiable. The expression profiles of these genes are presented in Fig. 5. Six out of the 13 characterized genes show a switching pattern where expression is significantly lower in F2 conditions but elevated during fasting (Fig. 5a), of these five are mainly related to energy metabolism (TMEM234, HAO2, CMBL, MMADHC, GYS2) and one is mainly related to immune function (F11). These genes were typically unaffected by the CR treatment. The remaining seven genes (Fig. 5b-c) showed the opposite pattern of elevated expression during feeding but reduced expression in fasting, of these two are mainly implicated in cell proliferation (ODC1, DUSP14), one is immune-related (IRF9; two transcripts) and one mainly involved in energy metabolism (ITPR3). The remaining three are involved in telomere elongation (SMG6), membrane transport (XKRX), and tubulin formation (TUBB2B). Most of these genes showed some level of reduced expression in CR compared to AL. A brief overview of the function of these genes is given in Additional Table 2.

**Fig. 6:**
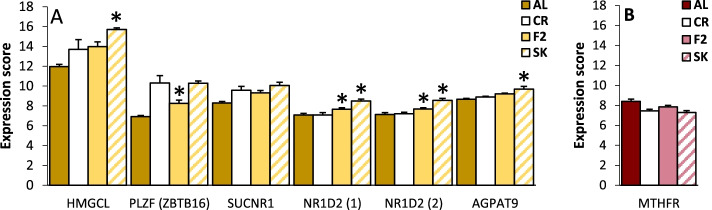
The expression of six genes showing both chronically changed and switching patterns under IF. This gene set is made up of (**A**) five genes that are up-regulated in fasting and (**B**) one gene that is down-regulated in fasting. The expression of these genes is significantly different from AL on both F2 and SK days in the same direction while F2 and SK expression are also significantly different from each other. * indicates significant difference from CR. Colors indicate which cluster each gene appeared in in the cluster analysis

**Fig. 7:**
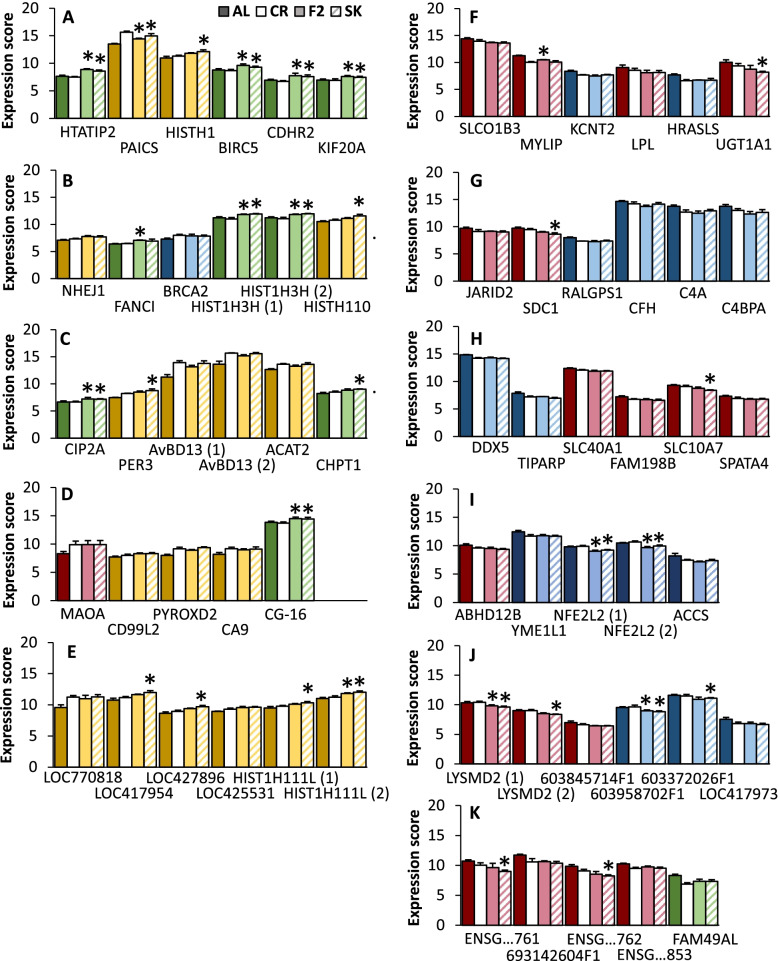
The expression of 54 genes showing a chronic change in gene expression under IF. The gene set is made up of (**A**-**D**) 21 characterized genes and (**E**) five uncharacterized genes which were chronically up-regulated in IF and (**F**-**I**) 22 characterized and (**J**) six uncharacterized genes which were down-regulated in IF. The expression of these genes is significantly different from AL on both F2 and SK days, but F2 and SK are not significantly different from each other. * indicates significant difference from CR. Colors indicate which cluster each gene appeared in in the cluster analysis

The chronic change criteria were met by a total of 70 genes, of which 49 were unique, identifiable and characterized (Fig. 1). Six of these correspond to the seven entries (i.e. one gene was represented by two transcripts) also showing a switching pattern, and the remaining 43 genes were non-switching. Five of the six genes showing both chronic change and a switching pattern were chronically upregulated compared to AL (Fig. 6a), four of these are mainly related to energy metabolism (HMGCL, SUCNR1, NR1D2 (two transcripts) and AGPAT9) and one mainly to cell proliferation (PLZF). All of these showed a switching pattern with higher expression in fasting conditions, while the only gene that was down-regulated compared to AL (MTHFR, Fig. 6b) was also further down-regulated in SK. The function of these genes is briefly summarized in Additional Table 3. The remaining 43 genes were fairly evenly split into 21 characterized (Fig. 7a-d**)** and five uncharacterized genes (Fig. 7e) that were upregulated and 22 characterized (Fig. 7f-i) and six uncharacterized genes (Fig. 7i-j) that were down-regulated compared to AL controls. The upregulated gene set was made up of 13 cell proliferation genes (HTATIP2, PAICS, HISTH1, BIRC5, CDHR2, KIF20A, NHEJ1, FANCI, BRCA2, HIST1H3H (two transcripts), HISTH110, CIP2A, CA9), two energy metabolism genes (ACAT2, CHPT1), one circadian gene (PER3), one immune-related gene (AvBD13; two transcripts), one monoamine oxidase (MAOA), one adhesion molecule (CD99L2), one potential cytoskeletal effector (PYROXD2) and one negative growth factor (CG-16). The downregulated gene set was made up of five energy metabolism genes (SLCO1B3, MYLIP, KCNT2, LPL, HRASLS, UGT1A1), three cell proliferation genes (JARID2, SDC1, RALGPS1), three immune-related genes (CFH, C4A, C4BPA), two circadian genes (DDX5, TIPARP), two transport proteins (SLC40A1, SLC10A7), one Golgi protein (FAM198B), one cell motility gene (SPATA4), two deaminating proteins (ABHD12B, ACCS), one mitochondrial metabolism gene (YME1L1) and one antioxidant response transcription factor (NFE2L2, two transcripts). The functions of these genes are briefly described in Additional Table 4.

**Fig. 8:**
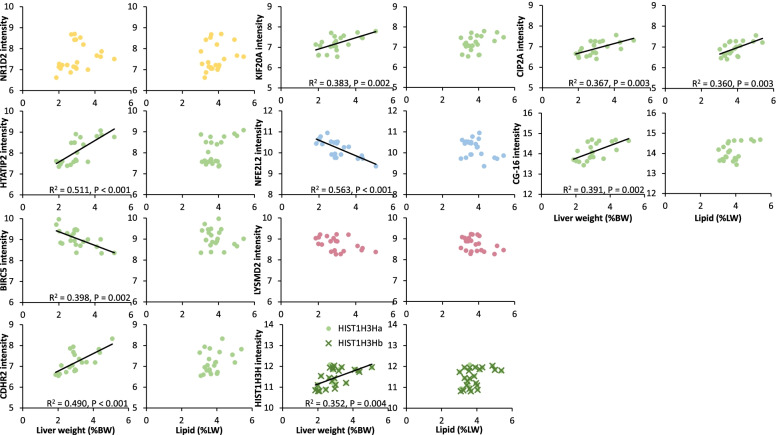
Correlation of the ten highlighted genes compared to relative liver weight and hepatic lipid concentration. These genes show chronically changed gene expression levels compared to both AL and CR. As for the cluster correlations, they were compared against seven physiological traits and the gene expression of three arcuate nucleus appetite-related genes, but only the correlations shown with a trendline here showed a significant correlation at *P* < 0.005 (Bonferroni-corrected from *P* < 0.05). Colors indicate which cluster each gene appeared in in the cluster analysis

Of the 49 genes whose expression levels were chronically affected by IF, ten were chronically changed compared to both AL and CR (Fig. 7a-e, i). The expression of each of these genes were correlated against the same traits of liver physiology and appetite regulation expression in the ArcN as in the cluster analysis (Fig. 8). The expression of HTATIP2, CDHR2, KIF20A, HISTH3H, CIP2A, CG-16, NFE2L2 and BIRC5 were significantly correlated with the relative mass of the liver (expressed as %BW) with only the last two mentioned showing negative correlations. The expression of CIP2A was also significantly and positively correlated with the hepatic total lipid concentration. No other significant correlations were found.

## Discussion

This study is the first to report large-scale transcriptomics of the liver’s response to long-term IF in chickens. In a similar study using an acute one-time fasting challenge, changes in hepatic gene expression consisted mostly of down-regulation in the fasting chickens [19]. In this study, however, we find similar numbers of up- and down-regulated genes across treatments, which may reflect that the birds had acclimated to their feeding treatments and did not perceive them as an acute challenge. A relatively even distribution of up- and down-regulated genes has also been reported in chicken adipose tissue following an acute fast [20] and in the liver of mice after acclimation to IF [21].

### Gene clusters

While we have focused solely on the liver in this study, our expectation is that the liver represents the bulk of the transcriptomic changes experienced under metabolic challenges. It has been previously shown that different diets lead to changed expression patterns in the liver, but not in muscle or adipose tissue [16]. Many of the genes whose expression levels were altered by different diet composition (high vs low fat) in that study appear in Clusters 2 and 4 in the current study, i.e. the clusters most clearly distinguishing between fed and underfed states. Of the genes overlapping between that study and this one, almost half (48%) appear in our Cluster 4, although the genes highlighted in the paper mostly appear in Cluster 2. Of their highlighted “fatty acid metabolism transcription factors” only one was differentially expressed in our study (JUN) and appeared in Cluster 4. When comparing the cluster analysis reported here to transcriptomic effects previously reported in acute fasting in both chickens and mammals, we notice that most overlap is usually seen with Cluster 2, which adds support to this representing a set of genes sensitive to underfeeding [20, 22, 23]. Food-deprivation immediately after hatching, however, shows transcriptomic effects mostly overlapping with Cluster 4. This is probably because Cluster 4 is heavily enriched in carbohydrate metabolism genes, a form of metabolism which is very limited before first feeding in hatchlings [24].

### Gene pathways

As expected, most of the observed differences in gene expression in the current study are related to energy metabolism, with Clusters 2 and 4 especially showing the switching pattern between fed and fasted days in the IF group. The KEGG pathway analysis identified these clusters as largely containing lipid vs carbohydrate metabolism genes, which goes well with the physiological processes known to be involved in metabolic switching and fasting. Cluster 1 was identified from the pathway analysis as largely amino acid metabolism and also shows some switching, but mainly appears to differentiate the CR birds from the other groups. What stands out as more interesting are the wide-spread effects on cell cycle transcriptomics, which are mostly clustered into Cluster 3. The expression patterns of Cluster 3 genes generally suggest increased hepatocyte proliferation in IF chickens while showing reduced expression in the growth-matched CR group. While this effect is especially clear in the fed IF birds where nutrient availability is expected to be at its highest, it’s interesting to note that it remains generally high compared to CR birds even during fasting. Transcriptomic signs of increased cell proliferation potential have previously been reported in the liver of chickens experiencing a one-time 48 h fasting challenge [19]. Our results suggest that this is indeed an important response to both long- and short-term fasting. This also agrees with what has been reported in rodents. For example, acute fasting for 48 h in rats has been shown to lead to increased cell proliferation rates after refeeding [25] and mice fed different IF diets show transcriptomic effects promoting improved brain function and disease resistance as well as increased neural proliferation after sham surgery or an induced stroke [26, 27]. In mice this pro-proliferative effect has been linked to lower levels of circulating leptin, but this association appears unlikely in chickens where leptin does not appear to have an endocrine function [28] and may suggest that a more conserved part of the metabolic switch, probably related to the switching between synthesis/storage and release of fatty acids or possibly to ketogenesis, is likely to be driving the effects on proliferation as these are some of the key metabolic roles of the liver [18, 24, 29].

### Important IF genes

Our expression pattern analysis highlighted 10 genes as particularly important as their expression levels were chronically changed in IF (ie. in both F2 and SK) compared to both AL and CR conditions, even though IF and CR treatments showed equivalent body weights. In general, the expression of these genes showed no or poor correlation with the liver physiology traits and ArcN appetite regulatory expression, suggesting that they add physiological information that the other traits we have recorded do not reflect. Similar to the cluster analysis, none of the genes showed expression that was significantly correlated with body mass. Of the 10 genes, six are related to cell proliferation namely HTATIP2, BIRC5, CDHR2, KIF20A, HIST1H3H and CIP2A. All six of these genes showed chronic upregulation in IF compared to AL and CR. Protein expression of HIV-1 Tat interactive protein 2 (HTATIP2) is known to be correlated with increases in triglyceride accumulation and decreases in fatty acid oxidation in mice [30]. This gene appears to be key to down-regulate glycolysis and oxygen consumption in glucose-limited conditions [31], and also functions as a redox sensor that induces apoptosis in response to oxidative stress [32]. While HTATIP2 can promote apoptosis, BIRC5 is an apoptosis inhibitor that promotes cell cycle progression in the liver [33]. Liver-specific knockout of survivin isoform 3 (BIRC5) reduces the regenerative capacity in mouse liver after surgery [34], seemingly opposite to the effects of IF reported by Manzanero et al. [26]. Kinesin family member 20A (KIF20A), histone cluster 1 H3 family member H (HIST1H3H) and cell proliferation-regulating inhibitor of protein phosphatase 2A (CIP2A) are all vital to cell cycle progression with KIF20A being involved in mitotic abscission [35], HIST1H3H being a replication-dependent histone specifically expressed during S phase [36] and CIP2A knockdown known to significantly increase the proportion of non-cycling cells [37]. Upregulation of HIST1H3H has previously been reported in human PBMCs after a 36 h fast [38]. In slight contrast to the other proliferation-related genes, cadherin-related family member 2 (CDHR2) is a contact inhibition trigger, leading to reduced proliferation rates when cell confluency is high [39, 40]. In total, the increased expression of these six genes should lead to a more fine-tuned plasticity of IF livers, allowing them to quickly adapt to varying availability of energy substrates in general and glucose especially. This kind of improved proliferative control also appears to be the case in mice on IF feeding schedules which show reduced cellular aging in the liver with reductions in both proliferation and apoptosis suggesting improved cellular maintenance rather than increased turnover of cells [41], lower levels of neural cell death with less dysfunctional neurogenesis after a stroke [26] and upregulation of pathways involved in cellular plasticity [27]. A somewhat similar functional pattern was seen in human PBMCs where a cluster of genes connected to cyclin-dependent kinase inhibitor 1A (CDKN1A) were affected by fasting. CDKN1A is known to block cell cycle progression under conditions of cellular stress and thus also acts as a form of sensor to fine-tune cellular plasticity [38]. Several of the genes clustering with CDKN1A in that study appear in our Cluster 2. CDKN1A is known to block cell cycle progression under conditions of cellular stress and thus also acts as a form of sensor to fine-tune cellular plasticity [22]. Several of the genes clustering with CDKN1A in that study appears in our Cluster 2.

### IF and inflammation

Of the remaining chronically affected genes, three may be related to inflammatory processes. The only upregulated gene among them is galectin CG-16, a chicken galectin that uniformly binds to chicken lymphocytes and has been shown to reduce the T cell response by 41–55% [42]. In mammals, galectins are known to induce T cell apoptosis and are important for down-regulation of the immune response after inflammation [43]. Nuclear factor erythroid-derived 2-like 2 (NFE2L2) can also decrease inflammation and acts as a transcription factor to protect cells from oxidative damage [44] but was chronically down-regulated in the IF chickens in this study. Increased oxidative damage has been previously reported in conjunction with increased respiratory capacity in the liver of IF-fed rats suggesting that this gene may warrant further attention [45]. The opposite pattern of increased NFE2L2 expression has previously been reported in broiler chickens experiencing cold stress [46] or fed high levels of poly-unsaturated fatty acids [47]. The expression of LysM domain containing 2 (LYSMD2) was also downregulated in IF chickens compared to both AL and CR. Although the actual function of the gene is yet unknown, it appears to be related to myeloid cell-mediated inflammation in humans [48] and has been found to be overexpressed in mice with fatty liver-induced hepatocellular carcinomas [49]. In dogs undergoing surgery for congenital portosystemic shunts, low hepatic expression of NFE2L2 during surgery is a predictor of poor recovery [50]. The expression of these three inflammation-associated genes could indicate either a reduced need or a reduced capacity for inflammatory responses in IF-fed birds. At this point we cannot say which of these two explanations is more likely, but it is worth noting that IF leads to a modest increase in heterophil-to-lymphocyte ratios (heterophils are the avian equivalent of neutrophils in mammals) which does suggest an immune activation under these conditions [10]. While the birds in our study were not subjected to any inflammatory challenge, it’s worth noting that IF mice show down-regulation of inflammatory genes after an ischemic stroke [27]. Whether this represents a difference between mammalian and avian physiology or is a sign that the down-regulation of inflammation-associated genes in this study is indeed a sign of reduced need for such proteins remains to be elucidated by future research. It is however worth noting that another mouse study found that immune-related genes whose expression was affected by fasting also quickly reduced to normal expression levels upon re-feeding [51].

### IF and the circadian rhythm

Finally, the expression of nuclear receptor subfamily 1 group D member 2 (NR1D2) was chronically upregulated in IF livers compared to both AL and CR treatments. This gene links circadian rhythm to hepatic lipid metabolism and may also have effects on cell proliferation. NR1D2 encodes one of the two Rev-erb protein subtypes which achieve the circadian patterns in protein and lipid metabolism [52]. The two subtypes are almost entirely overlapping in function which is typical for clock genes [52]. Rev-erb deficiency in mice is associated with mild hypoglycemia and elevated levels of free fatty acids after a 12 h fast with minimal change in circulating ketones [52]. Systemic double knockout mice show fragmented activity throughout the day [53]. NR1D2 is also overexpressed in some cancers, where it is important for focal adhesion formation. Knockdown of NR1D2 in these cells reduced cell size and viability, suggesting effects on cell proliferation [54]. The upregulation of NR1D2 in IF-fed chickens in this study may thus also contribute to a highly flexible liver that is well acclimated to the varying energy conditions typical of this feeding regimen. The chronic upregulation of a circadian regulator is quite expected, as hepatic circadian regulators are involved in diurnal regulation of hepatocyte size and has been suggested to play a similar role in IF [55]. It is also well known that the timing of feed intake has profound effects both on the entrainment of circadian rhythm and on the effects of IF [56–58]. Although we only see a few circadian rhythm genes showing significantly different expression between treatments in this study, that may be a result of the relatively short time frame used as similar time frames have proven too short to induce these effects in rodents [29].

## Conclusions

Intermittent fasting is well studied in both chickens and rodents, but few studies have tried to answer the question of whether the health benefits of IF observed in mammals are also present in poultry. We have shown that the chicken liver exhibits large and chronic changes in gene expression profiles under IF regimens that do not show clear correlation with apparent liver physiology and that differ from expression profiles in chickens experiencing daily feed restriction following equivalent growth curves. Most interesting is a general upregulation of genes involved in cell proliferation, and we argue that this should contribute to a highly flexible regulation of cell growth in the liver of IF-fed animals that potentially improves responsiveness to fluctuations in energy status and may as a consequence improve resistance to tissue damage. This agrees with rodent studies showing improved organ health and cellular maintenance in IF [14, 27, 56], implying highly conserved effects of metabolic switching despite differences in the hormonal environment between species. This also suggests that IF studies in poultry, which are already common in the form of “skip-a-day” studies, may provide valuable outgroup information to better understand the species differences between humans and rodents which are the most used experimental animals in fasting studies, e.g. by suggesting that fasting-induced increases in cell proliferation is not necessarily leptin-dependent. All in all, the results of this study will be useful to improve the understanding both of the physiology of intermittent fasting in general and the impact of intermittent fasting (“skip-a-day”) feeding regimens on farmed chickens.

## Methods

### Animal management and dissections

The Red Jungle Fowl used in this study came from a research population maintained at Linköping University, with its origins in Thailand (see [59] for details). The birds were raised indoors in pens of 260 × 120 cm on a 12:12 L:D light cycle. Temperatures were maintained at 26.8 ± 0.6 °C with a humidity of 30.6 ± 1.1% for AL and CR, and 28.2 ± 1.0 °C with 19.5 ± 0.6% humidity for IF. The provided feed (Penna, Lantmännen Lantbruk, Malmö) contained 18.4% crude protein amd 2.8% crude fat and was offered ad libitum until day 14, at which point it was switched to one of three feeding regimens as previously described in Lees et al. [13]. These consisted of the following: ad libitum feeding (AL, *n* = 48), chronic feed restriction to first 60% (days 14–24) and then 75% (day 25 onwards) of the age-matched AL food intake corrected for body weight (CR, *n* = 48), or intermittent fasting (IF, *n* = 43) in which birds were fed 150% of their age-matched and body weight-corrected AL intake daily on two consecutive days followed by fasting on the third day. Water was always offered ad libitum. Feed restriction was eased somewhat in the CR group from 25 days of age to maintain similar growth trajectories as the IF group. Any uneaten food was removed from the pen at mid-day (12:00) and quantified as previously described in [13]. Treatments were assigned to the birds pre-hatch, with hatching groups used as experimental groups. As only 24 arrays were available on the microarray, no replicates of treatments were conducted and individual animals were considered biological replicates and therefore the statistical unit for analysis. At the end of the experimental period, birds were killed by decapitation. Ad libitum and CR birds were sampled at 36 days of age and IF birds sampled either at 40 days of age on the second consecutive day of feeding (F2, *n* = 16) or at 41 days of age on a fasting day (SK, *n* = 16). Livers were immediately excised, weighed, frozen in liquid nitrogen and stored at -80 °C until the time of sample preparation. To minimize temporal effects on liver physiology, all animals used for this study were dissected in the 16:00–17:00 time frame (4–5 h after feeding). Animals were picked at random from the pen, with some animals excluded to achieve an even sex balance. Data on liver lipids and glycogen as well as ArcN gene expression have previously been published as part of a larger dataset in [13], where the assays used are also described in detail. All experimental handling of the animals, including the method of euthanasia, were approved by the Regional Council for Ethical Licensing of Animal Experiments (“Linköpings djurförsöksetiska nämnd”) which is the local equivalent of an IACUC.

### Sample preparation and microarray analysis

Upon thawing of samples, RNA was isolated and used to synthesize labeled cRNA for subsequent microarray analysis. Liver samples (~ 30 mg) were homogenized with a FastPrep® -24 (MP Biomedicals, USA) before total RNA isolation using TRIzol (Invitrogen). RNA was eluted using 30 μl of RNase-free water (Ambion, USA) and the samples analyzed for RNA quantity and quality (RNA Integrity Numbers) using a NanoDrop® ND-1000 (Thermo Scientific, USA), and a Bioanalyzer® instrument (Agilent Technologies, USA) respectively.

Once all RNA samples passed the quality control (RIN ≥ 8), they were converted to Cyanine 3-CTP labelled cRNA using one-color Low Input Quick Amp Labeling Kit (Agilent Technologies, USA), following the manufacturers’ protocol. In short, 100 ng RNA per sample was mixed with 10,000-fold diluted Spike-in Mix providing a positive control for the hybridization. Samples were mixed with T7 Primer and denatured at 65 °C for 10 min followed by 5 min incubation on ice. A master mix containing reagents for cDNA synthesis was added to all samples followed by a 2 h incubation at 40 °C. The enzymes were inactivated by a 15 min incubation at 70 °C before addition of a transcription master mix. The samples were then incubated for 2 h at 40 °C in order to synthesize complementary RNA whilst simultaneously labeling it with Cyanine 3-CTP. All samples were then purified using a RNeasy Mini Kit (QIAGEN, Germany) following the manufacturers’ protocol. After NanoDrop quantification of the samples, they were hybridized to SurePrint G3 Custom 8 × 60 K microarrays (Agilent Technologies, USA) overnight. This specific microarray design includes 2–3 probes each for a total of 20,771 probe sets corresponding to 16,360 annotated genome locations as previously described in [60] and has been previously validated by qPCR [61]. All arrays were scanned on an MS200 Microarray scanner (Roche NimbleGen, USA), and data was extracted via the Feature Extraction Software v12.0 (Agilent Technologies, USA). Two out of the 24 arrays failed quality control and were removed from the analysis, both from the F2 condition, leaving us with sample sizes of *n* = 6 for the AL, CR and SK treatments and *n* = 4 for the F2 treatment.

### Data processing

Expression data were analysed using the Bioconductor package, limma for R. Microarray data were checked by comparing logarithmic box plots of expression signals and by performing principle component analysis of the arrays. A linear modelling approach was used to detect differentially expressed probe sets. To adjust for multiple testing, Benjamini–Hochberg p-values were applied before determining statistical significance. Probe sets showing a significant expression difference (*p* < 0.01) greater than 1.4-fold were included in the subsequent analysis. Clusters of probe sets showing similar expression patterns across the four feeding treatments were identified by two-way hierarchical cluster analysis using the hclust function in R. Probe IDs for each cluster were then converted to Ensemble gene IDs and then annotated for KEGG pathways using DAVID online Functional Annotation Tool. Overviews on gene function were based on the entries for human orthologs on genecards.org (a summary based primarily on Entrez Gene, UniProtKB/SwissGene and GeneWiki) as well as chicken-specific descriptions from UniProt where available.

## Supplementary Information

**Additional file 1: Figure 1. **Venn diagrams showing the number of differentially expressedgenes in different treatment comparisons. **Figure 2. **Physiological measurements of the birds included in the study. **Table 1. **The number of up- and down-regulated genes when comparing expression between the groups. **Table 2. **Details on the 14 named genes showing a switching pattern. **Table 3.** Details on the six genes showing a switching pattern with chronically changed expression. **Table 4.** Details on the 45 named genes showing a chronically changed expression pattern.

## Data Availability

Microarray data has been uploaded to Array Express (http://www.ebi.ac.uk/arrayexpress) under accession E-MTAB-7829.
