# Supplementary material for: Intermittent fasting induces chronic changes in the hepatic gene expression of Red Jungle Fowl (Gallus gallus)
Source: BMC Genomics. 2022 Apr 14;23:304. doi: 10.1186/s12864-022-08533-5 (PMC9009039; doi:10.1186/s12864-022-08533-5)
Supplement: Supplementary file 1 — Additional file 1: Figure 1. Venn diagrams showing the number of differentially expressedgenes in different treatment comparisons. Figure 2. Physiological measurements of the birds included in the study. Table 1. The number of up- and down-regulated genes when comparing expression between the groups. Table 2. Details on the 14 named genes showing a switching pattern. Table 3. Details on the six genes showing a switching pattern with chronically changed expression. Table 4. Details on the 45 named genes showing a chronically changed expression pattern. [file 12864_2022_8533_MOESM1_ESM.docx]

Additional materials

**
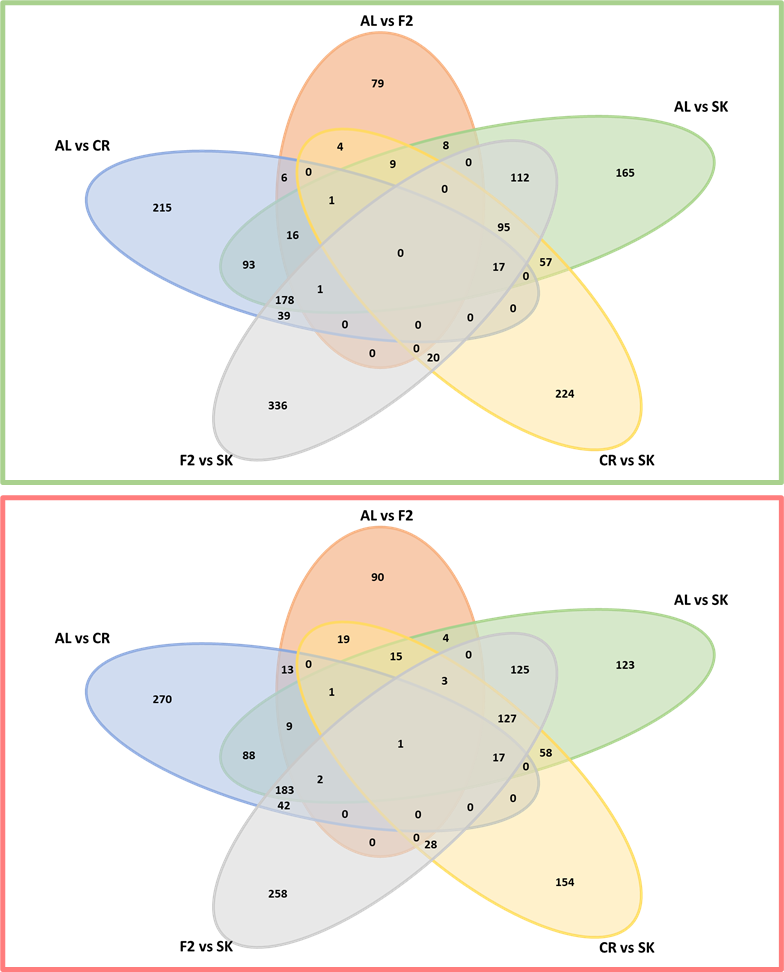
**

**Additional figure 1. Venn diagrams showing the number of differentially expressed genes in different treatment comparisons.** The top diagram shows the number of up-regulated genes and the bottom diagram the number of down-regulated genes.

**
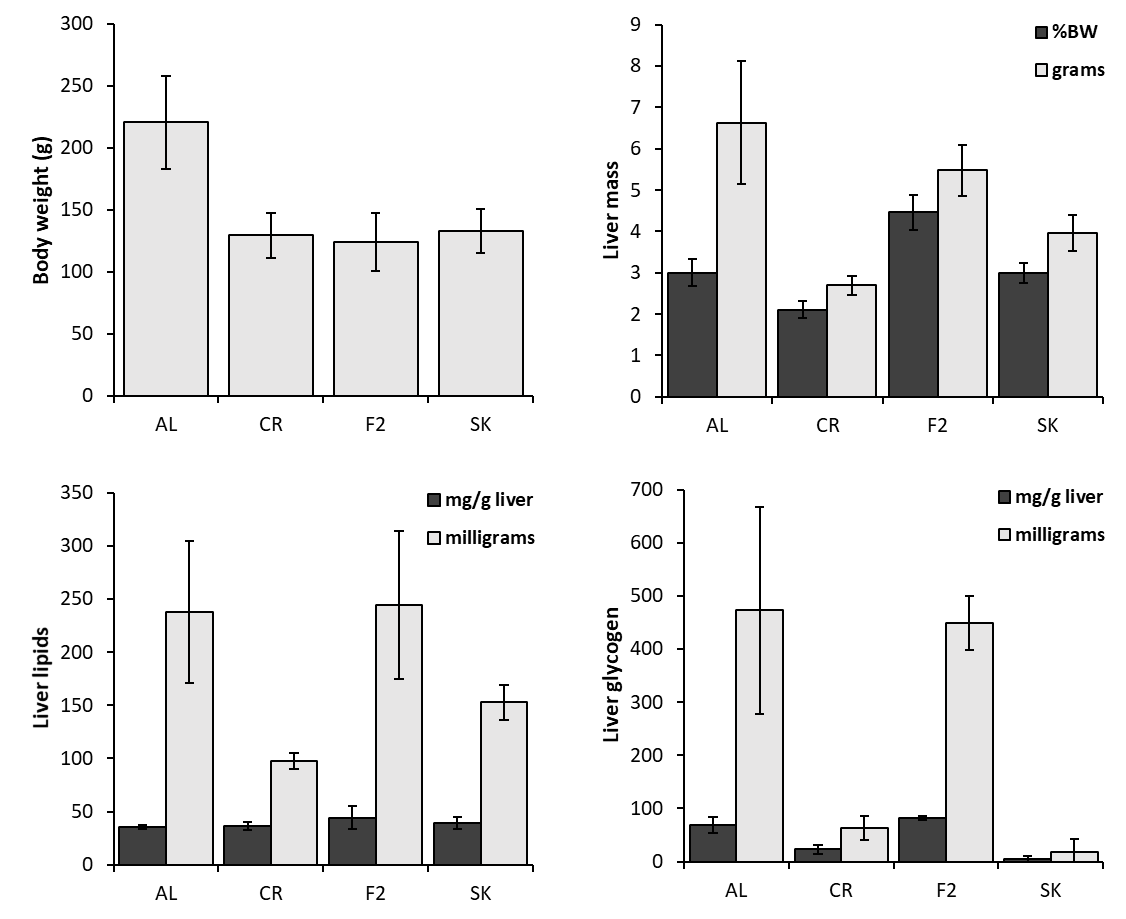
**

**Additional figure 2. Physiological measurements of the birds included in the study.** As previously reported (Lees et al 2017), AL birds were significantly larger than the other treatments at cull. They also had larger livers in absolute terms, but F2 livers were the largest when corrected for body weight. Total liver lipids varied largely between the treatment, but lipid concentration did not. For liver glycogen, both the total amount and the concentration varied between treatments.

**Additional table 1. The number of up- and down-regulated genes when comparing expression between the groups.**

|  | Up-regulated | Down-regulated |
| --- | --- | --- |
| AL vs CR | 566 | 626 |
| AL vs F2 | 124 | 157 |
| AL vs SK | 752 | 756 |
| CR vs SK | 427 | 423 |
| F2 vs SK | 798 | 786 |

**Additional table 2. Details on the 14 named genes showing a switching pattern.** Functional descriptions are based on the human orthologs and chicken-specific UniProt entries where available.

| **Abbreviation** | **Full name** | **Transcript** | **UniProt** | **Description** |
| --- | --- | --- | --- | --- |
| **ITRP3** | inositol 1,4,5-triphosphate receptor type 3 | ENSGALT00000004981 | F1N9Y1 | Receptor mediating intracellular Ca2+ release; key role in exocrine secretion underlying energy metabolism and growth |
| **SMG6** | nonsense-mediated mRNA decay factor | ENSGALT00000005046 | F1NNP8 | Component of telomerase ribonucleoprotein complex, promotes telomere elongation (also ribosome maturation) |
| **DUSP14** | dual-specificity phosphatase 14 | ENSGALT00000008755 | (retired) | Inactivates MAP kinases, related to "hypertrophy model" and "TGFbeta pathway" on Pathcards. |
| **IRF9** | interferon-regulatory factor 9 | NM_204558 |  | Transcription factor mediating type I interferon signaling, phosphorylates STAT1/2 |
| **XKRX** | XK-related, X-linked | ENSGALT00000010722 | F1P2I0 | Component of XK/Kell complex in Kell blood group system, membrane transporter |
| **CMBL** | carboxymethylenebutenolidase homolog | ENSGALT00000021277 | E1BXS8 | Cysteine hydrolase, highly expressed in liver cytosol |
| **GYS2** | glycogen synthase 2 | ENSGALT00000021664 | E1BXK3 | Liver glycogen synthase, rate-limiting step in glycogen synthesis |
| **F11** | coagulation factor 11 | ENSGALT00000022090 | F1NUN5 | Coagulation cascade protein, present in plasma as zymogen |
| **HAO2** | hydroxyacid oxidase 2 | ENSGALT00000023816 | E1C0E1 | Peroxisomal oxidation of 2-hydroxy-palmitate and L-alpha-hydroxy-acids in general |
| **TMEM234 (C1orf91)** | transmembrane protein 234 | ENSGALT00000035025 | (retired) | Member of drug/metabolite transporter superfamily |
| **TUBB2B** | tubulin beta 2B class IIb | NM_001004400 | P32882 | Beta isoform of tubulin, the major component of microtubuli; critical for axon guidance centrally and peripherally |
| **MMADHC (C2orf25)** | methylmalonic aciduria and homocystinuria, cbID type | NM_001008477 | Q57KP2 | Mitochondrial protein involved in B12 metabolism (DNA synthesis, myelin synthesis) |
| **ODC1** | ornithine decarboxylase 1 | NM_001167766 | F1NKA3 | Polyamine synthesis, essential for cell proliferation. Enzyme activity varies in response to growth-promoting stimuli. |
| TCP11L2 (twice) | T-complex 11-like 2 | NM_001001897 |  | uncharacterized |

**Additional table 3.** **Details on the six genes showing a switching pattern with chronically changed expression.** Functional descriptions are based on the human orthologs and chicken-specific UniProt entries where available.

| **Abbreviation** | **Full name** | **Transcript** | **UniProt** | **Description** |
| --- | --- | --- | --- | --- |
| **HMGCL** | 3-hydroxymethyl-3-methylglutaryl-CoA lyase | ENSGALT00000006455 | F1NWD1 | Mitochondrial HMG-CoA lyase, involved in leucine degradation and ketogenesis. |
| **MTHFR** | methylene tetrahydrofolate reductase | ENSGALT00000007342 | E1BXL1 | Converts a co-substrate for homocysteine remethylation to methionine. |
| **PLZF (ZBTB16)** | zinc finger and BTB domain containing 16 | ENSGALT00000011346 | (retired) | Nuclear protein involved in cell cycle progression; also transcriptional repressor that interacts with histone deacetylase. |
| **SUCNR1** | succinate receptor 1 | ENSGALT00000016875 | F1NBC7 | G-protein coupled receptor for succinate; involved in the development of hematopoietic progenitor cells. |
| **AGPAT9** | glycerol-3-phosphate acyltransferase 3 | NM_001031145 | Q5ZLL8 | Triacylglyceride (TAG) synthesis: converts glycerol-3-phosphate into lysophosphatidate. |
| **NR1D2** | nuclear receptor subfamily 1 group D member 2 | NM_205205 | (retired) | Nuclear hormone receptor; transcriptional repressor; involved in circadian rhythm; heme-dependent; regulates hepatic lipid metabolism via APOC3. |

**Additional table 4.** **Details on the 45 named genes showing a chronically changed expression pattern.** Functional descriptions are based on the human orthologs and chicken-specific UniProt entries where available.

| **Abbreviation** | **Full name** | **Transcript** | **UniProt** | **Description** |
| --- | --- | --- | --- | --- |
| **ABHD12B** | abhydrolase domain containing 12B | ENSGALT00000032445 | F1NMM5 | A paralog of this plays a key role as lysophosphatidyl serine lipase in CNS and regulates endocannabinoid signaling |
| **ACAT2** | acetyl-CoA acetyltransferase 2 | NM_001039287 | F1NT2O | Cytosolic acetoacetyl-CoA thiolase, involved in lipid metabolism |
| **ACCS** | ACC synthase-like protein 1 | ENSGALT00000012985 | F1NR60 | Does not synthesize ACC, but deaminates L-vinylglycine |
| **AvBD13 (GAL13)** | avian β-defensin 13 | NM_001001780 | QVIV18 | Bactericidal protein |
| **BIRC5?** | survivin isoform 3 (retired annotation) | ENSGALT00000014178 | (retired) | Promotes cell proliferation and prevents apoptosis, more active in fetal development than in adults |
| **BRCA2** | BRCA2, DNA repair associated | ENSGALT00000036458 | F1P3B2 | Homologous recombination and double-strand break repair, potentially in S phase checkpoint activation |
| **C4A** | complement C4A | ENSGALT00000027531 | F1P587 | Part of classical activation pathway, mediator of local inflammation |
| **C4BPA** | C4b-binding protein, α-chain precursor | NM_001033642 | F1P2M6 | Controls classical complement activation pathway |
| **CA9** | carbonic anhydrase 9 | ENSGALT00000034416 | F1NIF0 | Reversible hydration of CO2; a role in cell proliferation has been suggested |
| **CD99L2** | CD99 molecule-like 2 | ENSGALT00000014767 | F1NGN9 | Adhesion molecule during leukocyte extravasation (by similarity), potential cell aggregation molecule |
| **CDHR2** | cadherin related family member 2 | ENSGALT00000005196 | F1NLE0 | Ca2+-dependent cell-cell adhesion, forms complex with CDHR5 |
| **CFH** | complement factor H | ENSGALT00000003831 | E1C7P4 | Regulates complement activation, restricts innate defense to microbes |
| **CG-16** | galectin CG-16 | NM_206905 | A0A1D5PBG4 | Cell-cell and cell-matrix interactions; autocrine negative growth factor |
| **CHPT1** | choline phosphotransferase 1 | ENSGALT00000020835 | R9PXP4 | Phosphatidylcholine synthesis from CDP-choline (CEPT1 paralog) |
| **CIP2A (KIAA1524)** | cell proliferation regulating inhibitor of protein phosphatase 2A | NM_001030785 | F1NEH4 | Important oncogene, inhibits tumor suppressor PP2A; promotes anchorage-independent cell growth |
| **DDX5** | DEAD-box helicase 5 | NM_204827 | A0A1L1RUB6 | Transcriptional co-regulator; component of PER complex |
| **FAM198B (C4orf18)** | family with sequence similarity 198, member B | ENSGALT00000015333 | E1BY05 | Golgi protein with unknown function |
| **FANCI** | Fanconi anemia complementation group I | NM_001114851 | F1P1Z1 | Double-strand break repair by homologous recombination, S and G2 checkpoints |
| **HIST1H3H** | histone cluster 1 H3 family member h | ENSGALT00000019209, ENSGALT00000031955 | P84229 | Highly expressed during S phase and in liver |
| **HISTH1** | histone H1 | ENSGALT00000019187 | F1NPJ2 | Linker part of histone complex |
| **HISTH110** | histone cluster 1, H1.10 | ENSGALT00000019199 | P02286 | Histone 1 |
| **HRASLS** | HRAS like suppressor | ENSGALT00000011646 | E1C3C1 | Ca2+-dependent phospholipase acting on phosphatidylcholine and phosphatidyl ethanolamine |
| **HTATIP2** | HIV-1 Tat interactive protein 2 | ENSGALT00000004067 | A0A1L1RLC7 | Tumor suppressor, inhibits nuclear import; may act as redox sensor; related to "cytoskeleton signaling" |
| **JARID2** | jumonji and AT-rich interaction domain containing 2 | NM_001012862 | F1NWZ0 | Transcriptional repressor and negative regulator of cell proliferation signaling, essential during embryonic development of liver and heart |
| **KCNT2** | potassium channel, Na-activated subfamily T member 2 | ENSGALT00000003864 | (retired) | Rapidly activates outward rectifier K+ currents when high Na+ and Cl-; hepatic ABC transporter, related to diabetes and cholesterolemia |
| **KIF20A** | kinesin family member 20A | NM_001012783 | Q5ZKH9 | Required for CDC-mediated cytokinesis; microtubule transport |
| **LPL** | lipoprotein lipase | NM_205282 | P11602 | Hydrolysis of triglycerides from chylomicrons and VLDLs, coactivated by APOC2; expressed in heart, muscle and adipose |
| **MAOA** | monoamine oxidase A | NM_001030799 | A0A1D5PZV1 | Oxidative deamination of amines such as dopamine, serotonin and adrenaline |
| **MYLIP** | myosin regulatory light chain interacting protein | NM_001012561 | Q5F429 | Sterol-dependent inhibitor of cellular cholesterol uptake and LDLR degradation; cytoskeletal effector |
| **NFE2L2** | nuclear factor erythroid-derived 2-like 2 | NM_205117 | F1P315 | Transcription factor that regulates antioxidant response genes |
| **NHEJ1** | non-homologous end joining factor 1 | ENSGALT00000018498 | F1NVP8 | DNA repair factor, preferentially double-stranded breaks |
| **PAICS** | phosphoribosylaminoimidazole carboxylase and phosphoribosylaminoimidazolesuccinocarboxamide synthase | NM_205524 | R9PXP8 | Purine biosynthesis |
| **PER3** | period circadian clock 3 | ENSGALT00000000797 | E1C8E2 | Core component of circadian clock although redundant with PER1 and PER2 |
| **PYROXD2** | pyridine nucleotide-disulphide oxidoreductase domain 2 | ENSGALT00000011349 | F1N6K9 | “Probable oxidoreductase", protein interactions known in mammals may suggest cytoskeleton effects |
| **RALGPS1** | Ral GEF with PH domain and SH3 binding motif | ENSGALT00000001330 | E1C5G4 | May be involved in cytoskeleton organization |
| **SDC1** | syndecan 1 | ENSGALT00000026589 | F1NV24 | Links cytoskeleton to interstitial matrix; required for HIV-1 Tat internalization; cell binding, signaling, proliferation and migration; regulates exosome biogenesis |
| **SLC10A7** | solute carrier family 10 member 7 | NM_001031131 | Q5ZJH8 | Symporter; sometimes referred to as sodium/bile acid transporter 7 but does not transport bile acids |
| **SLC40A1** | solute carrier family 40 member 1 | NM_001012913 | Q5F3K3 | Iron export from duodenal epithelium |
| **SLCO1B3** | solute carrier organic anion transporter family member 1B3 | ENSGALT00000021478 | G4XPB1 | Liver-specific transporter for uptake up molecules that are excreted with bile acids and bilirubin (e.g. estradiol, T3, DHEAS, methotrexate, taurocholate, leukotriene) |
| **SPATA4** | spermatogenesis associated 4 | NM_001031138 | Q5XLA5 | Cilium-dependent cell motility |
| **TIPARP** | TCDD inducible poly(ADP-ribose) polymerase | ENSGALT00000016682 | F1N973 | Histone modifier; possibly involved in T-cell function; CLOCK-related? |
| **UGT1A1** | UDP-glucuronosyltransferase | ENSGALT00000006673 | F1P1M1 | Transforms small lipohilic molecules such as bilirubin into excretable forms |
| **YME1L1** | YME1 like 1 ATPase | NM_001031512 | A0A1D5P7R8 | Mitochondrial protein metabolism and maintenance of mitochondrial structure |
| HIST1H111L (HIST1H1C) | histone H1.11L | ENSGALT00000019221, NM_001040643 | | pseudogene |
| FAM49AL | family with sequence similarity 49 member A-like | ENSGALT00000006094 | E1C8F4 | uncharacterized protein |
